# Increasing the power of interpretation for soil metaproteomics data

**DOI:** 10.1186/s40168-021-01139-1

**Published:** 2021-09-29

**Authors:** Virginie Jouffret, Guylaine Miotello, Karen Culotta, Sophie Ayrault, Olivier Pible, Jean Armengaud

**Affiliations:** 1 Université Paris-Saclay, CEA, INRAE, Département Médicaments et Technologies pour la Santé (DMTS), SPI, F-30200 Bagnols-sur-Cèze, France; 2 Laboratoire des Sciences et de l’Environnement (LSCE-IPSL), UMR 8212 (CEA/CNRS/UVSQ), CEA Saclay, Université Paris-Saclay, Orme des Merisiers, F-91191 Gif-sur-Yvette, France; 3 Laboratoire Innovations technologiques pour la Détection et le Diagnostic (Li2D), Université de Montpellier, F-30207 Bagnols-sur-Cèze, France

**Keywords:** Bioinformatics, Cascaded search, Database, Interpretation, Metagenomics, Metaproteomics, Microbiome, Soil, Tandem mass spectrometry

## Abstract

**Background:**

Soil and sediment microorganisms are highly phylogenetically diverse but are currently largely under-represented in public molecular databases. Their functional characterization by means of metaproteomics is usually performed using metagenomic sequences acquired for the same sample. However, such hugely diverse metagenomic datasets are difficult to assemble; in parallel, theoretical proteomes from isolates available in generic databases are of high quality. Both these factors advocate for the use of theoretical proteomes in metaproteomics interpretation pipelines. Here, we examined a number of database construction strategies with a view to increasing the outputs of metaproteomics studies performed on soil samples.

**Results:**

The number of peptide-spectrum matches was found to be of comparable magnitude when using public or sample-specific metagenomics-derived databases. However, numbers were significantly increased when a combination of both types of information was used in a two-step cascaded search. Our data also indicate that the functional annotation of the metaproteomics dataset can be maximized by using a combination of both types of databases.

**Conclusions:**

A two-step strategy combining sample-specific metagenome database and public databases such as the non-redundant NCBI database and a massive soil gene catalog allows maximizing the metaproteomic interpretation both in terms of ratio of assigned spectra and retrieval of function-derived information.

Video abstract

**Supplementary Information:**

The online version contains supplementary material available at 10.1186/s40168-021-01139-1.

## Background

Soil hosts complex microbial ecosystems which are crucial for numerous ecosystem services, including plant growth and animal life [71]. These ecosystems can be affected by anthropogenic pressure and climate change [29]; therefore, it is important to understand their structure and how they function [6]. Due to the broad diversity of components they include and their dynamic relationships, soil microbial ecosystems are complex by nature [17]. Indeed, soils are open systems exposed to highly variable environmental parameters such as temperature, hygrometry, gas, metal, and chemical contaminants, which can influence microbial populations and their functions. Thanks to improved meta-omics technologies, the number of in-depth molecular studies of soil environments is increasing [58]. Since the pioneering metagenomics works almost two decades ago, molecular phenotyping approaches such as metatranscriptomics, metaproteomics, and meta-metabolomics have emerged and been used to attempt to understand how these systems function at various levels. Specifically, metaproteomics allows the identification and quantification of proteins, which are the workhorses of the cells, and can be used to monitor more integrated levels, such as pathways and general functions [56, 70]. Humic acids and potential contaminants may interfere with protein extraction, thus metaproteomics methods must be specifically developed to suit each soil type [32, 63]. Despite these difficulties, several pioneering studies have been performed on soils extracted from forests [40, 74], arid environments [7], agricultural areas [39, 50], permafrost [27], and from mining drainage [53]. Sediments — deposited material arising from weathering, erosion, and transport processes — also contain complex microbial ecosystems [19, 64].

Metaproteomics involves protein extraction and trypsin proteolysis, detection of the resulting peptides by tandem mass spectrometry, interpretation of MS/MS spectra to assign peptide identities, and higher-level interpretation in terms of taxonomy and function [34, 49]. MS/MS spectra acquired in metaproteomics studies are interpreted by comparison to a database listing the sequences of all the proteins potentially present in the sample. To create such a database, the most appropriate strategy is to perform metagenomics or metatranscriptomics on the same sample. These databases can then be translated (in six- or three-frames) to derive the theoretical protein sequences. Alternatively, protein sequences from the organisms identified in similar samples can be compiled for complementing metagenomics information [27, 75]. Another alternative is to assemble a specific database based on the organisms identified after 16S rRNA amplicon sequencing and taxonomical assignment [73] or potentially present in the habitat where the sample was obtained [9]. The choice made between read- or contig-based databases may influence the identification rate. For animal metaproteomics, a contig-based database has been shown to be the most productive strategy [62]. However, if the necessary metagenomics information is not available, generalist databases such as NCBInr or UniProtKB/Swiss-Prot can also be used [24]. Despite these multiple options, the large diversity and dynamic range of taxa contained in some samples, such as soils and sediments, represents a true challenge for metaproteomics interpretation and limits protein identification [58, 70]. Indeed, this diversity results in a search space for metaproteomics databases that is naturally much larger than that required for single-organism proteomics. To counteract the negative effects of an inflated database size on sensitivity and accuracy of peptide-to-spectrum matching (PSM), several strategies have been proposed. These include database reduction using a two-step search [28], where matches derived from the first search — performed without false discovery rate (FDR) threshold — are used for a second search round, during which a stringent threshold is applied. This type of cascaded search was successfully implemented to define the metaproteome of the gut microbiota from a sentinel, non-sequenced animal [20], and lichen-associated bacterial communities [11]. These databases are protein-centric, i.e., focused on the main proteins across all clades, and can thus successfully highlight the main functions at play within the most abundant microbial organisms from the ecosystem sampled.

Soil/sediment metaproteomics is currently challenging because a large proportion of organisms in soil samples have yet to be taxonomically characterized [47] and only a small fraction of reference genome sequences are available in public data repositories. Furthermore, the community structure of such samples may vary dramatically over time and space. Although numerous large-scale metagenomics studies have been performed on soil samples [5, 51], the contribution of specific soil gene catalogs to improving metaproteomics interpretation has not yet been estimated. In this study, we recorded metaproteomics data from a soil core consisting of the annual sediment deposit in a floodplain which provides long-term records of particle-bound pollutants (metals, radionuclides, pharmaceuticals, and numerous persistent organic pollutants) released by the Seine River (France), including effluents from the Parisian megacity [1]. We tested several strategies when interpreting the metaproteomics data acquired for this soil sample. These strategies included sample-specific metagenomics data, a topsoil gene catalog constructed from a large diversity of sites [5], and genome sequences from reference microorganisms. We found that a significant increase in the numbers of MS/MS spectra interpreted and functionally annotated was obtained when a combination of all types of information was used in an appropriate cascaded search strategy. The results substantially improved our understanding of the soil microbiota.

## Results

### Benchmarking databases created from sample-specific metagenomics data

**Fig. 1:**
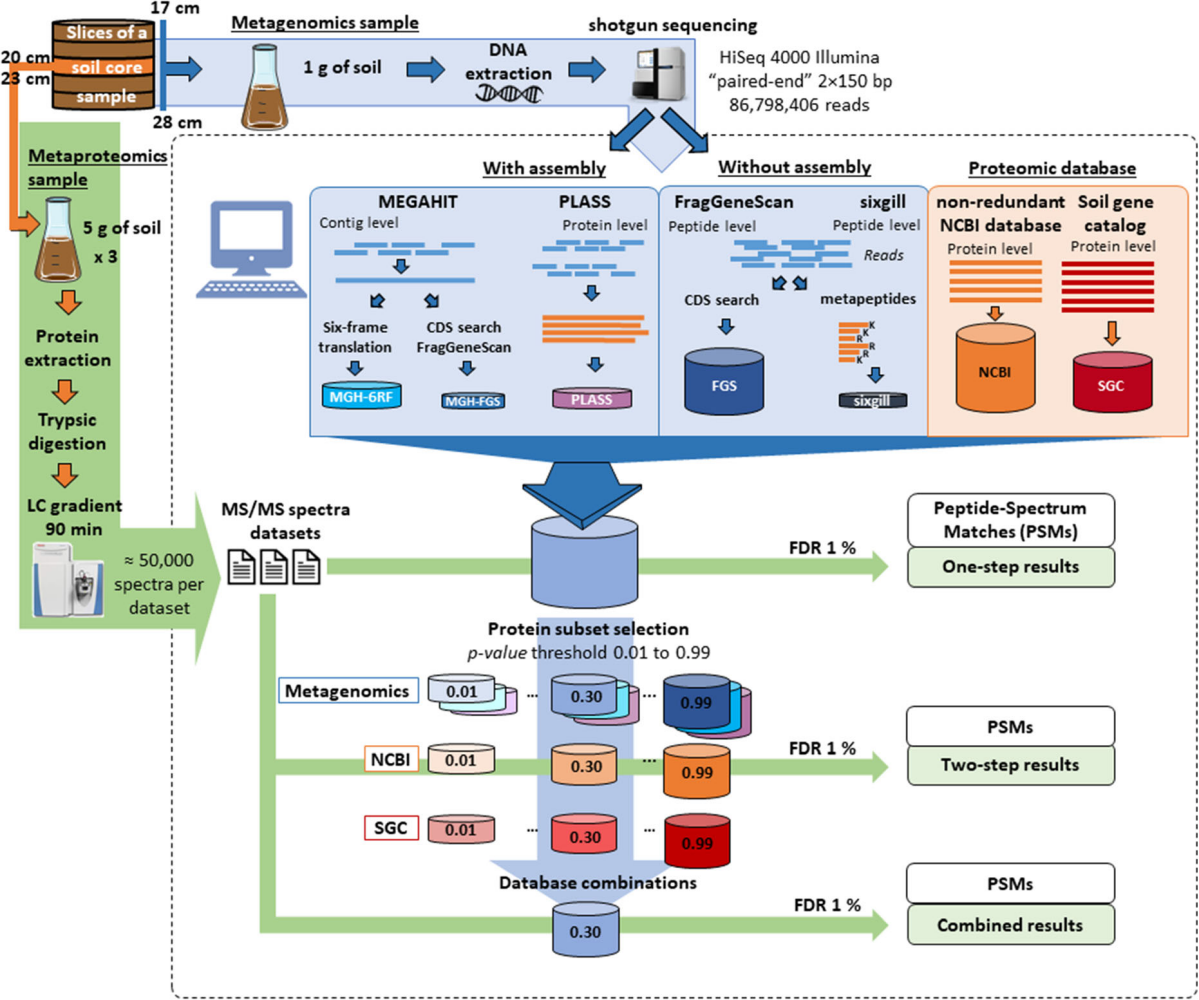
Workflows assessed for metaproteomics analysis of environmental samples. The left (green) and top (blue) arrows show the experimental steps tested to obtain MS/MS spectra and metagenomics reads. The dashed-line panel presents the bioinformatics analysis, with, at the top, database construction either from metagenomics data with or without assembly step, or from generic databases. The bottom part shows the process used to refine and enhance the attribution rate through multiple search rounds. A first analysis on full databases was used to select protein-based subsets at various *p*-values. New searches on these subsets then identified the databases performing best

Different databases built from metagenomics data acquired on a sediment sample were evaluated for metaproteomics based on the number of PSMs as main criterion. For this, a soil core was collected from the Seine River floodplain at the Bouafles site (France) located downstream of Paris. The 1-m core was cut up into 3-cm slices. A shotgun metagenome sequencing dataset comprising ~ 87 million Illumina paired-end reads was acquired for the slices corresponding to 17–28-cm depth in the soil core after extracting DNA from a pool of the five corresponding slices. Figure 1 shows the five options used to construct the sequence databases: (i) reads were assembled with MEGAHIT and the resulting contigs were translated in the six possible reading frames (MGF-6RF), (ii) selected based on coding gene sequences predicted by FragGeneScan tool (MGH-FGS), (iii) reads were assembled directly at the protein level using PLASS assembler (PLASS), (iv) coding sequences were selected from reads by FragGeneScan without assembly step (FGS), or (v) selected only tryptic peptides capable of undergoing tandem mass spectrometry, as intended by sixgill (sixgill). Table 1 indicates the number of sequences and size of the resulting databases. First, reads were directly assembled using the MEGAHIT tool, which has been benchmarked as one of the best assemblers [69], resulting in 972,629 contigs with 60.8% GC content, 939 N50, and 101,728 L50. The largest contig length was 48,284. A systematic six-reading-frame translation was used to produce the MGH-6RF database, which comprises almost 22 million possible protein sequences and a billion amino acid residues. To decrease the size of the database and remove erroneous polypeptide sequences, the FragGeneScan tool was then used to select predicted protein-coding sequences (CDS). The resulting MGH-FGS database is much more focused, retaining only 17% of the information contained in MGH-6RF. A third database was created by assembling reads at the protein level using the PLASS assembler. This strategy bypasses silent single nucleotide sequencing errors and compresses the possible single nucleotide polymorphisms that could occur across closely phylogenetically related strains present in the sample. It should be noted, however, that this tool may lead to chimeric assemblies between similar protein sequences. Application of the PLASS assembler resulted in a database containing 16 million proteins with a mean length of 112 amino acids, which is a significant increase in size (+ 80%) compared to the proteins listed in MGH-6RF. To avoid possible bias due to assembly of metagenome reads either at the nucleotide sequence level or at the amino acid sequence level, small truncated polypeptide sequences can be directly predicted from the short reads. The FragGeneScan tool performs this type of prediction and was used to produce the FGS database, which is three times larger than MGH-6RF. Finally, the sixgill algorithm was applied to directly produce a list of putative tryptic peptides amenable to tandem mass spectrometry which are well represented in at least two reads. The resulting sixgill database was rather small, representing only 8% of the size of MGH-6RF.

**Table 1:** Sample-specific metagenomic databases and generic databases

Databases	Tools used/database origin	Computational time^**a**^ (hours)	Size of the database (in residues)	Number of protein entries
MGH-6RF	MEGAHIT + six-frames translation	13	1,028,880,437	21,883,653
MGH-FGS	MEGAHIT + FragGeneScan	13	168,662,946	1,269,322
PLASS	PLASS	6	1,784,677,737	16,004,028
FGS	FragGeneScan	43^b^	2,939,955,188	72,130,656
sixgill	Sixgill	5.5	82,314,892	2,577,349
NCBI	Non-redundant NCBI	-	41,817,980,956	108,307,546
SGC	Soil gene catalog	-	21,962,323,955	159,657,012

^a^Computer used: 10 CPU, 240 Gb RAM memory

^b^Single thread

**Fig. 2:**
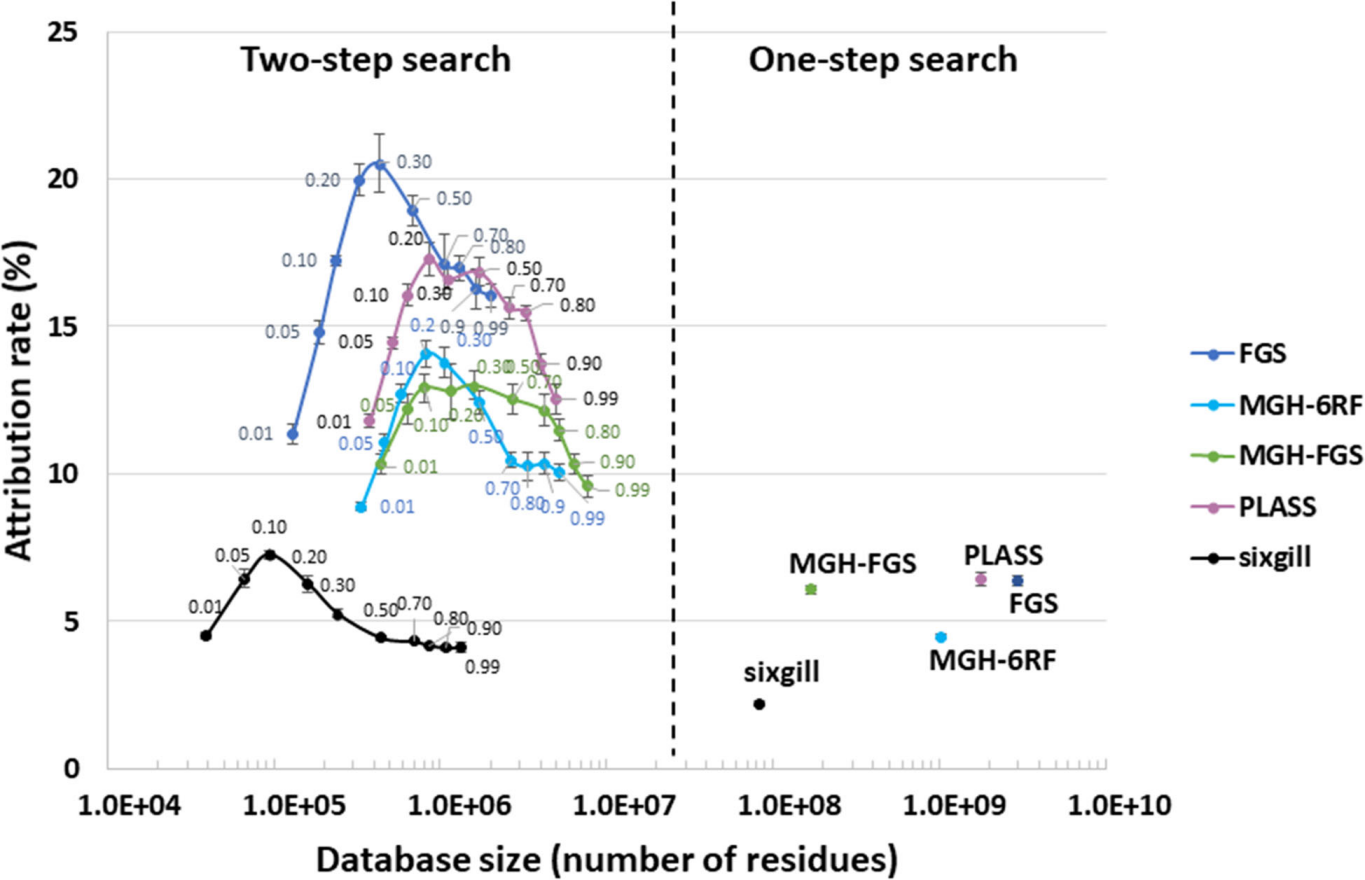
Attribution rates for MS/MS spectra, obtained with sample-specific metagenomics databases. Databases were generated using the following workflows: FragGeneScan only (FGS), MEGAHIT and FragGeneScan (MGH-FGS), MEGAHIT and six-frame translation (MGH-6RF), sixgill, and PLASS. The results obtained when using the complete databases are indicated on the right (one-step search), whereas the results obtained on refined databases containing proteins selected at distinct *p*-values during the first-round search are shown on the left. Searches were performed at decoy-free FDR 1% and results are ordered based on the database size, using a logarithmic X-axis. The small numbers indicate the *p*-value threshold used for each search. Three biological replicates were used to calculate the mean and standard deviation values.

Proteins were extracted from three equal aliquots of the same section of the soil sample (slice 20–23 cm of the sampled soil core). The peptides derived from the biological triplicates after trypsin proteolysis were analyzed by nanoLC-MS/MS, producing 59,501, 59,917, and 59,141 MS/MS spectra. These three datasets were subsequently used separately to estimate search variability across the different databases even if the biological samples taken for metagenomics and metaproteomics do not match perfectly. Figure 1 shows the two strategies used to interpret MS/MS signals with the five databases. First, databases were queried at the same 1% FDR in a one-step search strategy. Because decoy database searches are problematic for large database [15, 26] with increased occurrence of reversed peptide sequences corresponding to true peptide sequences and variability depending on how the decoy is constructed, we used a decoy-free FDR evaluation for this. As recommended by Jagtap et al. [28], a two-step database search strategy was also conducted. The first search round selected protein sequences at low stringency, whereas the second search performed with this sub-database validated the most relevant hits. In this case, several *p*-value thresholds (0.01, 0.05, 0.10, 0.20, 0.30, 0.50, 0.70, 0.80, 0.90, 0.99) were tested for the first-round search to estimate the impact of this parameter on the final results. The second search was performed at decoy-free 1% FDR. Figure 2 presents the results obtained following application of the two strategies, in terms of PSM attribution rate. The *X*-axis represents the size of the databases used in the final step of the cascaded search. Notably, for all conditions tested, the result variability estimated on the three experimental metaproteomic datasets was quite low, at less than 0.5% in most cases. The one-step search method allowed between 2.2 and 6.5% of MS/MS spectra to be assigned, with the maximum reached using the PLASS database. The sixgill database search performed poorly (only 2.2% MS/MS spectra assigned) even though it was the smallest, and theoretically the best-adapted to the proteomic data format. The two-step database search method significantly increased the proportion of MS/MS spectra assigned, with 3-fold higher values recorded for most conditions. Although this increase was expected, the results reveal that the improvement ratio depends strongly on the stringency of protein selection during the first identification round. Here, optimal *p*-values could clearly be identified for each database: 0.10 for sixgill, 0.20 for MGH-6RF and PLASS, and 0.30 for MGH-FGS and FGS. Using the two-step search method, higher numbers of confident PSMs were assigned, reaching at best 7.3% for sixgill, 13.0% for MGH-FGS, 14.1% for MGH-6RF, 17.3% for PLASS, and 20.5% for FGS. As with the one-step search, the two-step search strategy performed better with the FGS and PLASS databases, but a clear advantage was noted for the FGS database. Unexpectedly, among the sequencing-read-assembly strategies, a better attribution rate was obtained for PLASS compared to MEGAHIT. This result highlights the power and reliability of a strategy based on assembly of peptide sequences rather than nucleic acid sequences and demonstrates the added value of retaining variants that are discarded by the MEGAHIT algorithm. These results also show that predicting coding sequences after assembly (MGH-FGS) does not provide significant advantages over six-frame translation (MGH-6RF) in the two-step search method, as these databases allowed 13.0% and 14.1% MS/MS assignment, respectively. This result directly contrasted with that of the one-step search strategy, where 6.1% and 4.5% of MS/MS spectra were assigned, respectively. In conclusion, the highest attribution rate (20.5%) and coverage of the microbial metaproteome was obtained with the FGS database when queries were performed at *p*-value 0.30 in the first search round, using a large database with only short sequences (mean length, 40.7 amino acids).

### Assessing the potential of generic databases

**Fig. 3:**
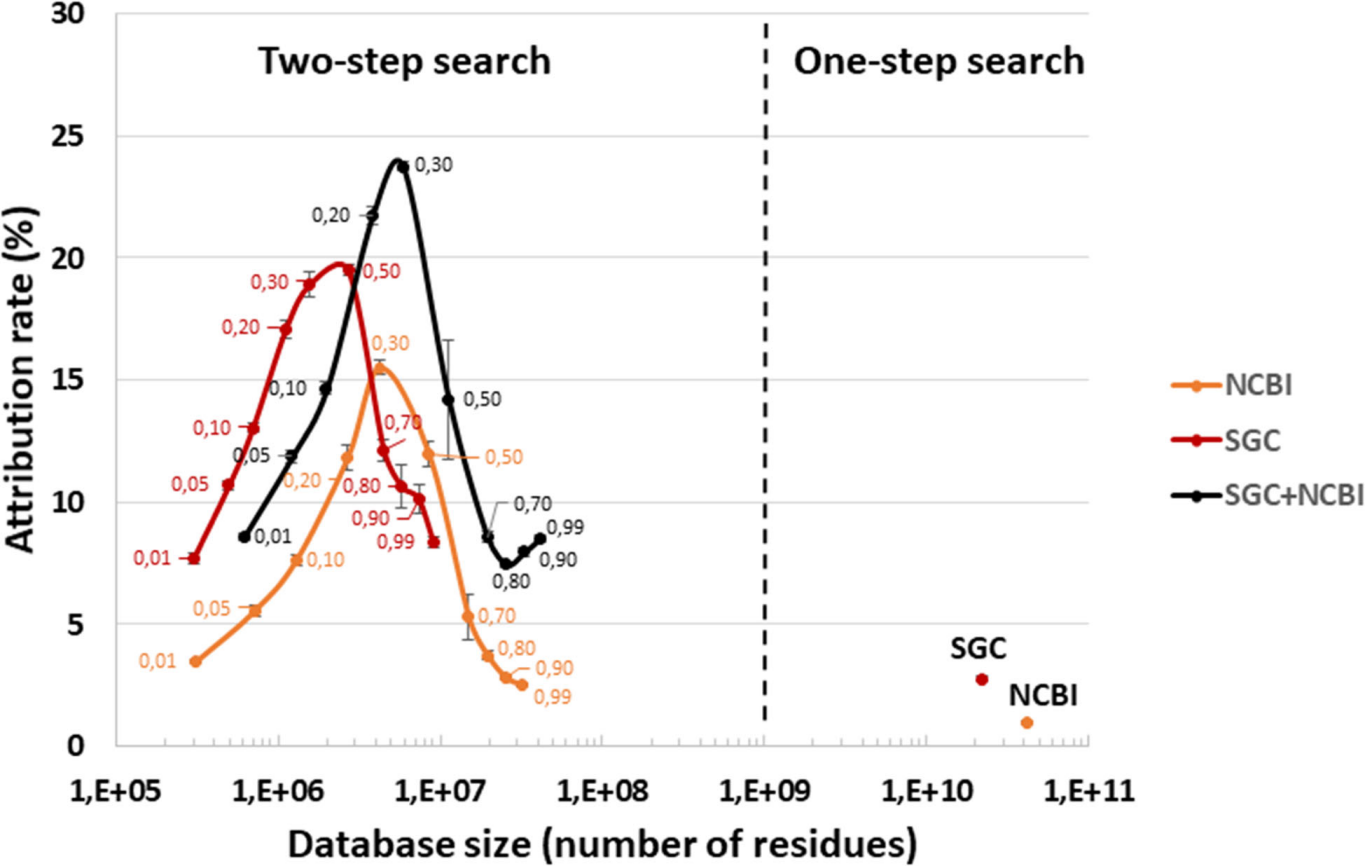
Attribution rates for MS/MS spectra when using generic databases. The results obtained with SGC and NCBI are shown as a function of the database size using a logarithmic *X*-axis. The results obtained with the whole databases are presented on the right (one-step search), whereas the results obtained on refined databases containing proteins selected at distinct *p*-values during the first-round search are indicated on the left (two-step search). The SGC+NCBI databases were obtained by merging the reduced NCBI and SGC protein databases at each *p*-value cutoff. Searches were performed at decoy-free FDR 1%. The small numbers indicate the *p*-value threshold used for each search. Three biological replicates were used to calculate the mean and standard deviation values.

As shown in Fig. 1, two generic databases were also used to interpret the three MS/MS datasets: the giant NCBInr database, totaling 41.8 billion residues and comprising the protein sequences from 27,137 species; and the Soil Gene Catalog (SGC) database compiling an extensive catalog of genes established by metagenomics of numerous topsoil samples [5]. This SGC database is twofold smaller than the NCBInr database. Figure 3 shows the results of the one-step and two-step search methods. The proportion of MS/MS spectra assigned at decoy-free FDR 1% with these two databases was low when used directly: 0.9% for NCBInr and 2.8% for SGC. Such a result was expected with the two generic databases, as the first one is not specifically representative of microorganisms likely to be present in soil samples, but also due to the huge size of the two generic databases, which hinders correct FDR estimates. Using the two-step search method, the ratio of assigned spectra increased significantly (Fig. 3). At the optimal *p*-value thresholds, SGC performed better than NCBInr, with 19.5% versus 15.5% of MS/MS spectra assigned to peptide sequences. Because the two databases could be complementary in terms of environmental sequence coverage, we also assessed the effect of merging SGC and NCBI sub-databases at various *p*-values (SGC+NCBI). As indicated in Fig. 3, the proportion of MS/MS spectra assigned was increased to 23.8% when using this combined database, for which the optimal *p*-value threshold was 0.30. Interestingly, this assignment ratio was higher than that obtained with the classical approach, consisting in nucleic acid sequence assembly and protein sequence prediction (MGH-FGS). These results suggest that, in the future, generic databases — which are continuously expanding to include new environmental metagenomics projects and NCBInr updates — could perform as well as sample-specific metagenomics databases, even when treating difficult environmental samples. This prospect would decrease the per-sample cost of metaproteomics analyses.

### Combining sample-specific metagenomics data and generic databases

We next went on to test the effect of a combination of sample-specific metagenomics databases and generic databases on the attribution rate for MS/MS spectra. The best-performing reduced databases from the two-step search strategy were selected: FGS_0.30, PLASS_0.30, MGH-6RF_0.30, NCBI_0.30, and SGC_0.30 (at the most common optimal *p*-value of 0.30). Table 2 reports the number of sequences and residues contained in these reduced databases. In addition, we created two new databases comprising only the peptides detected in the two-step search performed with the generalist databases, resulting in the NCBIp_0.30 and SGCp_0.30 databases. Table 2 shows the 16 combinations of databases tested in this new round of MS/MS interpretation, their sizes, and the assignment rate obtained at decoy-free FDR 1%. Combining the reduced FGS_0.30 and NCBI_0.30 databases for a single search resulted in an average of 24.9% of MS/MS spectra assigned for the three metaproteomic datasets. This proportion represents a significant increase compared to the optimal FGS_0.30 database (20.5%). Reduced FGS_0.30 and NCBIp_0.30 also performed well, with 24.8% spectra assigned, but a greater variability was noted. Use of the reduced SGC_0.30 and FGS_0.30 databases also resulted in a higher number of PSMs (25.9%) compared to FGS_0.30 alone. Concatenation of the FGS_0.30, SGC_0.30, and NCBI_0.30 sub-databases slightly improve results (26.2% MS/MS assignment). The same trend was observed with combinations of PLASS_0.30 and general sub-databases. Indeed, PLASS_0.30+SGC_0.30 (24.8%) performed better than PLASS_0.30+NCBI_0.30 (22.4%) and PLASS_0.30+SGC_0.30+NCBI_0.30 (24.6%). MGH-FGS_0.30+SGC_0.30 (24.4%) performed less than MGH-FGS_0.30+SGC_0.30+NCBI_0.30 (24.8%). The alternative MGH-6RF database performed slightly less with 23.4% combined with SGC_0.30 and 23.6% with SGC_0.30+NCBI_0.30. Decreasing the size of the merged database by selecting only the peptide sequences detected in a two-round search did not systematically increase the assignment rate, as shown when comparing PLASS_0.30+NCBIp_0.30 (22.1%) and PLASS_0.30+NCBI_0.30 (22.4%). In conclusion, our results demonstrated that, for experimental soil metaproteomics data, the assembly of metagenomics reads at either the nucleic acid (MGH) or the polypeptide level (PLASS) could be detrimental to the MS/MS spectrum assignment rate compared to direct use of reads (FGS). Here, the highest MS/MS spectral assignment rate was obtained when a sample-specific metagenomics database was combined with generalist databases in a two-round search strategy.

**Table 2:** Combining sample-specific metagenomic databases and generic databases

Combined databases	Size of the database (in residues)		Number of sequence entries		Attribution rate (%)	
	Mean	sd (%)	Mean	sd (%)	Mean	sd (%)
SGC_0.30 +NCBI_0.30	5,849,545	1.9	15,340	2.1	23.76	0.2
SGCp_0.30 +NCBIp_0.30	220,207	8.8	18,644	8.8	23.73	6.9
FGS_0.30 +NCBI_0.30	4,670,124	2.3	17,101	2.4	24.98	0.6
FGS_0.30 +SGC_0.30	1,990,818	1.7	18,073	2.5	25.94	0.4
FGS_0.30 +SGC_0.30 +NCBI_0.30	6,275,438	1.9	25,298	2.3	26.21	0.9
FGS_0.30 +NCBIp_0.30	525,294	2.6	18,878	2.5	24.84	1.1
FGS_0.30 +SGCp_0.30 +NCBIp_0.30	646,100	1.9	28,603	4.8	27.24	5.8
PLASS_0.30 +NCBI_0.30	5,351,198	2.1	15,541	2.0	22.42	1.2
PLASS_0.30 +SGC_0.30	2,673,152	1.8	16,512	2.2	24.83	0.6
PLASS_0.30 +SGC_0.30 +NCBI_0.30	6,957,772	1.8	23,738	2.1	24.62	0.5
PLASS_0.30 +NCBIp_0.30	1,206,368	2.2	17,318	2.2	22.05	0.7
PLASS_0.30 +SGCp_0.30 +NCBIp_0.30	1,327,067	1.2	27,042	5.2	25.42	4.7
MGH-FGS_0.30 +SGC_0.30	3,158,743	1.8	15,862	2.1	24.40	0.4
MGH-FGS_0.30 +SGC _0.30 +NCBI_0.30	7,443,363	1.7	23,088	2.0	24.83	0.8
MGH-6RF_0.30 + SGC _0.30	2,629,015	2.0	15,664	1.9	23.43	0.8
MGH-6RF_0.30 + SGC _0.30 +NCBI_0.30	6,913,635	1.9	22,890	1.9	23.58	0.4

Mean and standard deviation (sd) were calculated based on the three biological replicates

### Functional annotation with the optimal combined databases

**Fig. 4:**
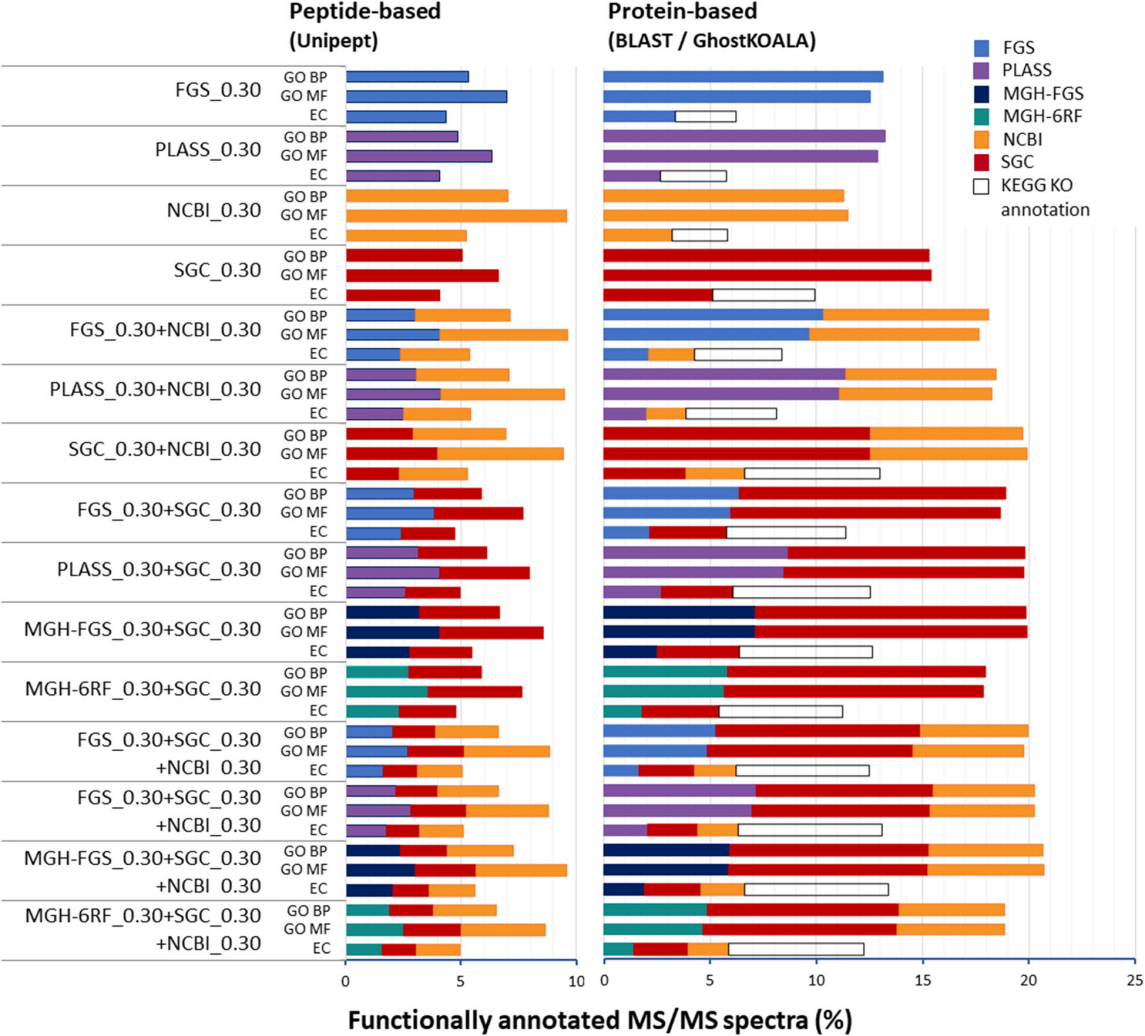
Functional annotation of peptides and proteins identified at FDR 1%. The databases used to identify proteins are indicated on the left. For each database, the percentage of PSMs annotated are indicated in terms of GO_BP, GO_MF terms, and EC numbers obtained either by Unipept or using Diamond BLASTp queries on Uniref50. In the latter case, the GO OWL tools were used to retrieve GO slim annotations; EC numbers and KO entries were retrieved using the GhostKOALA web-service. The grey squared areas on the EC lines represent the KEGG KO annotation level, from which EC numbers were extracted

As the aim of metaproteomics is to analyze the function of the proteins identified, we next assessed the levels of functional annotation obtained with the four databases performing best in terms of attribution rates, when used alone or in combination. Figure 4 shows the functional annotation obtained following three processes. First, peptides identified at FDR 1% using the FGS_0.30, PLASS_0.30, SGC_0.30, and NCBI_0.30 databases, and combined databases were annotated by applying the Unipept tool which is based on the lowest common ancestor approach. This peptide-based functional annotator returns molecular function (MF) and biological process (BP) Gene Ontology (GO) terms, and enzyme commission (EC) numbers. In parallel, identified proteins were annotated using GO slim level and KEGG Orthology (KO) terms by the Diamond BLASTP and GhostKOALA tools, respectively. Notably, here, Unipept annotation produced less annotated MS/MS spectra than Uniref50 BLASTP searches, suggesting that protein level functional annotation is more powerful than peptide level. In terms of databases, PLASS_0.30 and FGS_0.30 performed well, as judged using the Uniref50-based GO BP annotation, with 13.2% and 13.1% of MS/MS spectra functionally annotated, respectively (Fig. 4). Interestingly, PLASS_0.30 performed better at the functional level than at the attribution rate level. SGC_0.30 database performed better than NCBI_0.30 database with 15.3% and 11.3% of MS/MS spectra functionally annotated, respectively. For the four databases, between 64 and 81% of PSMs were functionally annotated. SGC_0.30+NCBI_0.30 performed better than individual metagenomics databases, with 19.7% of spectra annotated. The combination of metagenomics and generic databases was very efficient to improve the functional attribution rate compared to the standalone databases: the combinations of each standalone MGH-FGS_0.30, PLASS_0.30, and FGS_0.30 databases with SGC_0.30 and NCBI_0.30 databases allowed 20.6%, 20.3%, and 20.0% of spectra to be functionally annotated, respectively. Regarding the GhostKOALA-based KO annotation (Fig. 4), the same trend was observed, with maximized functional annotation obtained when using databases combining metagenomics and generic information. Thus, MGH-FGS_0.30+SGC_0.30+NCBI_0.30 and PLASS_0.30+SGC_0.30+NCBI_0.30 provided 13.4% and 13.1% of functionally annotated spectra, respectively, with a slightly higher contribution from soil gene catalog database. With the reduced SGC_0.30 and NCBI_0.30 databases, 9.9% and 5.8% of spectra were functionally annotated, respectively. When combined as SGC_0.30+NCBI_0.30, 13.0% of spectra were functionally annotated, representing 55% of PSMs for the database, with a higher contribution of SGC. In conclusion, the best result in terms of functional annotation was obtained when a sample-specific metagenomics database was combined with a generalist database and soil-specific database in a two-round search strategy. Remarkably, combined generic databases allowed 55% of PSMs to be functionally assigned when the different procedures tested were merged.

### Consistency of functional and taxonomic annotations

**Fig. 5:**
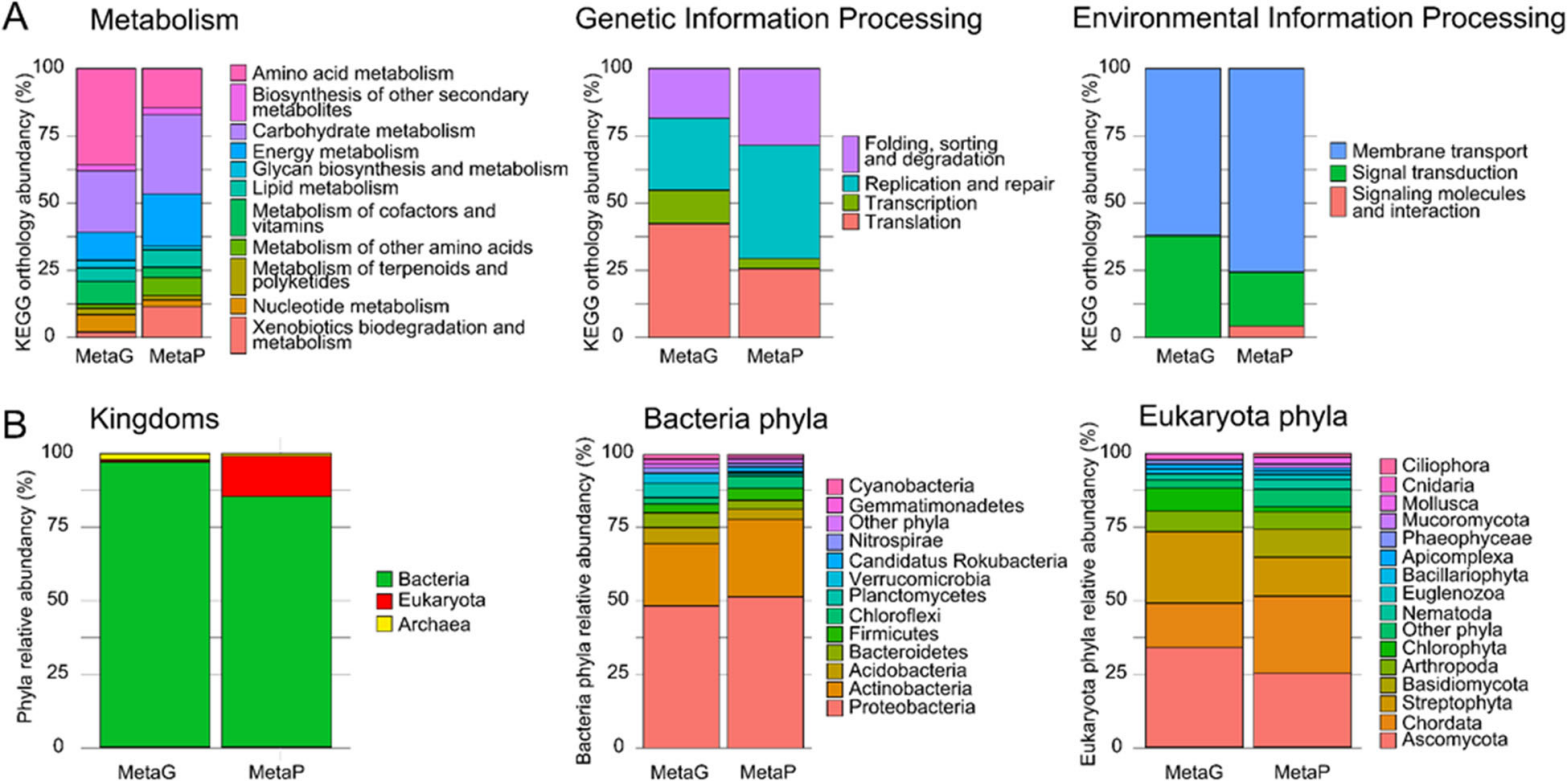
KO and taxonomical annotation of soil metagenomics data and metaproteomics data. Metagenomics data were annotated using the MG-RAST pipeline, with KO and taxonomic analysis. Peptides and sequences attributed at FDR 1% of the combined reduced sample-specific and generic database FGS_0.30+SGC_0.30+NCBI_0.30 were analyzed using Unipept and GhostKOALA to retrieve taxonomic and functional annotations, respectively. The PSMs for the three biological replicates were cumulated. **A** Functional annotation of three KEGG categories: metabolism (left), genetic information processing (center), and environmental information processing (right). A total of 16% of reads and 72% of PSMs were annotated in terms of function. **B** Taxonomic annotation of metagenomics data (MetaG) and metaproteomics data (MetaP) at Kingdom (left), Bacterial phyla (center), and Eukaryotic phyla (right) levels. Phyla represented less than 1% of the total were merged under “Other phyla”. A total of 17% of reads and 21% of PSMs were unequivocally annotated in terms of taxonomy

Metagenomics and metaproteomics results were compared in terms of functional annotation based on KO. For this comparison, metagenomics reads obtained for the soil sample were analyzed using the MG-RAST pipeline [44] to produce KO annotations. These results were compared to those obtained following GhostKOALA functional annotation for the metaproteomics dataset, which grouped together the three biological replicates, and was interpreted using the FGS_0.30+SGC_0.30+NCBI_0.30 database. Figure 5A shows three main functional groups: “metabolism”, “genetic information processing”, and “environmental information processing”. Remarkably, the different activities within each of these groups were relatively consistent when assessed by the two methodologies, even though they do not rely on the same molecules or measurements. In this soil sample, “amino acid metabolism”, and “carbohydrate metabolism” and “energy metabolism” were the most abundant functional categories according to metagenomics and metaproteomics data. Interestingly, metaproteomics allows us to descend deeper into the functional category “signaling molecules and interaction pathway”, as proteins classified as “signaling molecules and interaction” are identified. In contrast, this category is under-represented by the metagenomics analysis (Fig. 5A).

Metagenomics and metaproteomics datasets were also interpreted at the taxonomic level. As shown in Fig. 5B, the phyla identified and their ratios were consistent. At the domain level, both methodologies indicated a vast predominance of bacteria in the sample. Within this superkingdom, Proteobacteria and Actinobacteria are the most abundant phyla, but a large diversity of phyla were represented. Remarkably, both methodologies can highlight the presence of a candidatus phylum, namely *Candidatus Rokubacteria*, which was previously reported to predominate in Amazonian rainforest soil [35]. Some discrepancies were noted for the estimated Eukaryote ratio. Clearly, metagenomics underestimated the presence of eukaryotic cells compared to metaproteomics. However, this underestimation is expected as the volume of these cells is much higher than the volume of bacteria, whereas their nucleic acid molecule content is similar, leading to a higher ratio when protein biomass is measured compared to nucleic acid estimation. The eukaryotic phyla identified and their respective quantities are consistent between the two technologies, although the huge diversity present in the sample could have been a source of bias.

## Discussion

The aim of this study was to determine the database construction and search approach that would maximize the information extracted by metaproteomics analysis of soil sampled from a floodplain along the Seine River, downstream of Paris (France). Through the list of proteins it provides, and their abundances, metaproteomics brings a new dimension to the study of microbiota by delivering the list of organisms present in a sample and their respective biomasses [52], and by providing information on how the microbial community functions [12, 34]. Most metaproteomics interpretation pipelines up until now have been evaluated using human microbiome samples such as saliva [28] or feces [48, 62, 67] or laboratory-assembled microbial mixtures [61, 67]. As shown previously with these samples, the choice of the workflow in metaproteomics is critical as it controls the peptide identification. An average of 21% of attribution rate at FDR 1% was obtained with human fecal samples using a combination of the search algorithms X!Tandem and OMSSA against a customized protein sequence database containing 6 millions of proteins from different sources such as metagenomes, bacterial, and human genomes [49]. In comparison, less than 3% of spectra were assigned in our study with the SGC database which comprises 159 million of sequences. The sample preparation and mass spectrometry acquisition parameters are also critical as they may impact the attribution rate. Based on the same bioinformatics workflows, identification rates varying in the range 12 to 35 % were obtained at FDR 1% on a fecal sample using the same reference database (van der Bossche, Kunath et al. 2021). Because of its inherent characteristics, soil is a difficult matrix to work with for metagenomics and metaproteomics [58]. The extent of the diversity of microorganisms in soils is considered a significant bottleneck for the interpretation of omics data in general.

To construct the most appropriate database for use when interpreting metaproteomics data, it is generally recommended to use metagenomics data acquired for the same sample. From the sampled soil, ~ 87 million Illumina paired-end reads were recorded, corresponding to 13 Gbp of sequenced nucleotides. Whether this sequencing effort comprehensively represents the microbial community found in the sample is a key question. In some soil studies, the cumulated efforts made to analyze a large collection of samples is considerably greater. For example, a total of 730 Gbp of sequenced nucleotides were obtained for the analysis of soil communities in phosphorus-deficient and phosphorus-rich tropical soils [74]. Similarly, a total of 250 Gbp of sequenced nucleotides were recorded when reconstructing the microbial metabolic network in a host geological nuclear waste repository [4]. For the present study, we assumed that the depth of metaproteomics achieved with a standard analysis (here, a 90-min nanoLC-MS/MS run) would be relatively limited, and that the metagenomics information obtained should be sufficient to effectively represent the most abundant microorganisms. If the objective was to analyze the whole 1-m soil core, the metagenomics efforts would have to be multiplied, along with the monetary costs of nucleic acid sequencing, to produce a database representative of the whole core. Sequencing depth directly influences the outcome of any attempt to assemble metagenomics data, but more importantly, the use of short-read next-generation sequencing combined with long-read technology should also be taken into account in such projects [23]. Once again, the corresponding costs will be the main factor driving the implementation of these combined sequencing technologies for soil analysis, but we are confident that a combined approach could boost metagenomics-based metaproteomics.

The metagenomics reads obtained in this study were treated either with MEGAHIT, FragGeneScan, or sixgill. Our results using the five constructed databases confirmed that a strategy with two query rounds, as recommended for unusually large databases [28], performs better than direct assignment, whatever the database used. However, at least in our set-up, an optimum should be considered to select the entries used to create the sub-database. Indeed, a proteomics approach was recently used to assess the quality of transcriptomics data and their assembly [14], and metaproteomics data could be used in a similar way to assess the quality of metagenomics data assemblies.

With the datasets considered here, the best PSM attribution rate was obtained for the FragGeneScan CDSs predicted directly from trimmed reads (20.5%). As expected, this attribution rate was lower than those obtained with similar instruments when studying single organisms for which a well-annotated genome is available. For example, a rate of 61% PSM assignment was reported for the *Microbacterium oleivorans* A9 strain [18]. However, our rate it is quite similar to that reported for an animal proteogenomics study (21% [66];. The complexity of soil samples in terms of strains means that many possible peptide co-elutions and thus chimeric MS/MS spectra can be produced. We therefore expect that the rate of assignment would be further improved using higher-performance acquisition instruments.

The high quality of theoretical proteomes from isolates available in generic databases such as NCBInr and their large numbers advocate for use of these resources in metaproteomics interpretation pipelines. Indeed, the use of selected annotated genomes has previously been explored [13, 27, 50, 75], as has the use of the Uniref100 database [55] or the NCBInr database [30, 68, 72]. Here, two generic databases were assessed for their usefulness in interpreting the soil metaproteomics data: NCBInr and the SGC soil gene catalog. The two databases were complementary in terms of environmental sequence coverage, and the spectrum attribution rate of the combined database was 23.8%, which is higher than with a search against a sample-specific metagenomics database, but without the cost. Therefore, this strategy could be advantageous whenever numerous samples of diverse origins are to be analyzed.

Previous studies indicated that merging protein sequence databases from several samples might improve the peptide identification rate [59, 62]. Here, we combined metagenomics data analyzed with FragGeneScan, SGC, and generic database such as NCBI in a two-step search strategy. This approach produced the best assignment rate, with 26.2% of MS/MS spectra assigned. We therefore recommend this approach for use with other experimental metagenomics and metaproteomics datasets. Another previous study indicated that combining Uniprot with sample-specific metagenomics data could improve the number of peptides identified for samples from a biogas plant [25]. We found that the dedicated SGC database performed better than the generalist NCBInr database in the present study. Combining metagenomics sequencing data with data from a generic database could be performed while applying taxonomical constraints, as proposed previously [73]. However, this strategy is highly dependent on the presence of the identified organisms in the generic database and will consequently be sample-specific. Defining the optimal strategy in metaproteomics may depend on the research question to tackle as the objective may be either a focus on a few microorganisms with interesting metabolism, or the overall picture. In the first case, the design of a dedicated database emphasizing the genomes or metagenome-assembled genomes (MAGs) of interest may be well worth the effort required. In this vein, using the most abundant proteins identified by metaproteomics as guides to derive the taxonomic composition of the microbial community and expanding the search database with the genomes from the identified abundant species appears a promising two-stage strategy [57]. However, missing the identification of accessory proteins not present in the database could impact the understanding of the functionality of the microbial system. In the latter case, sequencing data allows MAGs binning, but a more globalized approach is often applied, either imposed by insufficient sequencing depth or preferred for speed, cost, sample, or resource availability. Taxonomical and functional assignation is then often performed at family or phylum levels using peptides, proteins, reads, genes, contigs, or scaffolds taxonomical and functional mapping. In that case, the assessment of metaproteomic databases can be performed using the PSM attribution yield.

Two significant criteria to consider when assessing the power of metaproteomics is how many of the peptides/proteins identified have taxonomy- and function-derived annotations. In metaproteomics, the taxonomical annotation is commonly performed with taxon-specific peptides using the lowest common ancestor approach, such as with the Unipept tool [21]. However, functional annotation works best at the protein level for metaproteomics, as shown here. The length of the sequences used to find a GO or KO has an impact on the percentage of PSMs functionally annotated. As shown here, peptide level functional annotation is improved using a sequence-based search for functional homologs at protein level, which both allows to annotate peptides missing in large protein databases (e.g., NCBI, Uniprot) and to enlarge the pool of proteins functionally associated with a given peptide, and thus the probability to gather GO or KEGG annotated proteins. Here, we found the optimal strategy in terms of both MS/MS attribution ratio and functional annotation ratio to be a combination of FGS, SGC, and NCBInr databases with 26.2% and 20.0% respectively. Combining SGC and NCBI databases results in a MS/MS attribution ratio of 23.8% and a functional annotation ratio of 19.7%. Therefore, this later strategy represents an interesting alternative for soil samples in the absence of sample-specific metagenomics sequencing data.

## Conclusions

In conclusion, combining sample-specific metagenomics data and generic databases in a two-step database search performed best for the soil sample analyzed in the present study, both in terms of ratio of assigned spectra and retrieval of function-derived information. Amalgaming a massive soil gene catalog and the generalist NCBInr database resulted in almost the same outcome. This result opens up broad prospects for the application of metaproteomics to soil samples, which includes a highly challenging matrix, as well as broad microbial diversity, and extensive complexity.

## Materials and methods

### Soil material

A soil core was sampled on May 23 2018 from a floodplain at Bouafles near the Seine River (France). The site has already been well characterized in terms of sedimentation and chemicals [2, 3, 37, 41]. The section of the core between 17 and 28 cm depth was sliced into five layers. Two grams of each layer were pooled and homogenized for DNA extraction. The mid-layer (20–23 cm depth) was used for protein extraction.

### DNA extraction from soil and sequencing

Soil DNA was extracted and sequenced by GenoScreen (Lille, France) from 1 g of lyophilized sample using an optimized protocol [65]. Briefly, soil was mixed with 100 mM Tris-HCl (pH 8), 100 mM EDTA (pH 8), 100 mM NaCl, 2% (w/v) polyvinylpyrrolidone (40 g/mol), and 2% (w/v) sodium dodecyl sulfate and subjected to bead-beating. DNA was precipitated with isopropanol, washed with 70% ethanol, and further purified using the MP Biomedials GeneClean Turbo kit (Fisher scientific). DNA libraries were constructed with the Nextera XT DNA Library Preparation kit (Illumina) and sequenced on a HiSeq 4000 Illumina run in 2 × 150 bp. Raw reads have been deposited in the Sequence Read Archive under dataset identifier SRX8818139, as part of Bioproject PRJNA648365. Reads were analyzed using the phylogenetic MG-RAST pipeline [44].

### Metagenomics analysis

Paired-reads were processed using the MEGAHIT workflow into the ASaiM Galaxy framework [8]. They were quality controlled and trimmed using FastQC and Trim Galore v0.4.3.1 with a Phred quality score cutoff of 20. MEGAHIT v1.1.2 [38] was used to assemble trimmed paired-reads into contigs with default parameters with a minimum kmer size of 21, maximum kmer size of 141, k-step of 12, and merge complex bubbles with length up to 20,098. The estimation of the assembly quality statistics was done with MetaQUAST [45] and the identification of potential assembly error signature with VALET. The percentage of unmapped reads were determined with Bowtie2 [36] and combined with MultiQC [16]. The MGH-6RF database was obtained by six-frame translation of the assembly, retaining only tryptic peptide sequences composed of at least five residues. PLASS [59, 60] was used with default parameters. Sixgill v0.2.4 [42] was used with the following parameters: minlength 10, minqualscore 30, minorflength 40, minlongesttryppeplen 7, and minreadcount 2. The paired-reads were processed with WHORMSS (Genoscreen) workflow consisting in demultiplexing and removing indexes in reads. The reads were trimmed and a Phred quality score cutoff of 30 was applied. The reads with a length lower than 75 bases were removed. Paired-reads were reassembled and low complexity sequences were removed as well as various contaminants including *Homo sapiens* sequences. FragGeneScan v1.3 [54] was applied with Illumina sequencing reads with about 0.01% error rate model to construct the FGS database.

### Soil gene catalog and NCBInr databases

The soil gene catalog [5] was downloaded from http://vm-lux.embl.de/~hildebra/Soil_gene_cat/ (accessed on 22 March 2021). NCBnr was downloaded from https://www.ncbi.nlm.nih.gov/ on 3 January 2018).

### Protein extraction and proteolysis

The proteins from 5 g of soil were extracted using the NoviPure Soil Protein Extraction Kit (Mo-Bio) as recommended by the supplier. After centrifugation, proteins from the 10-ml supernatant were precipitated by adding 2.5 ml trichloroacetic acid (50% w/v). Proteins were collected by centrifugation for 10 min at 6000×*g*. The resulting pellet was resuspended in 40 μL LDS 1X (Invitrogen) containing 5% beta-mercaptoethanol, sonicated for 5 min in an ultra sound bath and then heated to 99 °C for 5 min. Soluble proteins (25 μL per well) were subjected to SDS-PAGE gel electrophoresis on NuPAGE 4–12% Bis-Tris gel (Invitrogen) for 5 min at 200 V in MES/SDS 1X running buffer (Invitrogen). Proteins were stained for 15 min with Coomassie SimplyBlue SafeStain (Thermo Fisher Scientific), and then in-gel proteolyzed with trypsin gold (Promega) for 1 h at 50 °C, as recommended [22].

### NanoLC-MS/MS and interpretation

Peptides were analyzed on a Q-Exactive HF mass spectrometer (Thermo) coupled to an Ultimate 3000 nano LC system (Thermo), as described previously [33]. Tryptic peptides (8 μl) were desalted on a reverse-phase PepMap 100 C18 μ-precolumn (5 μm, 100 Å, 300 μm i.d. × 5 mm, Thermo) before separating peptides on a nanoscale PepMap 100 C18 nanoLC column (3 μm, 100 Å, 75 μm i.d. × 50 cm, Thermo) at a flow rate of 0.2 μL min^−1^ using a 90-min gradient of mobile phase A (0.1% HCOOH/100% H_2_O) and phase B (0.1% HCOOH/80% CH3CN). The gradient used was developed from 4 to 25% B in 70 min and then from 25 to 40% B in 20 min. The mass spectrometer was operated in Top20 data-dependent acquisition mode. Full MS scans were acquired from 350 to 1800 *m/z* at a resolution of 60,000 and the 20 most abundant precursor ions were sequentially selected for fragmentation with a dynamic exclusion time of 10 s. The resolution for the fragment scans was 15,000. Only ions with 2 or 3 positive charges were selected for fragmentation. MS/MS spectra were interpreted using Mascot Daemon software (version 2.6.1; Matrix Science) indicating 5-ppm tolerance for the parent ion and 0.02-Da tolerance for secondary fragments, 2+ and 3+ as possible peptide charges, a maximum of two missed cleavages, carbamidomethylation of cysteine as fixed modification, oxidation of methionine as variable modification, and trypsin as proteolytic enzyme. The FDR threshold was set at 0.01 using a decoy-free FDR method based on a mixture-model of four beta distributions which has been shown well adapted for handling large proteogenomics and metaproteomics datasets and databases [52]. The two-step database search strategy was initiated using several Mascot *p*-value thresholds (0.01, 0.05, 0.10, 0.20, 0.30, 0.50, 0.70, 0.80, 0.90, 0.99) for the first search round to select the protein sequences. The most time-consuming search (13 h) was noted for the first step NCBI database interrogation.

### Functional and taxonomic annotation and gene ontology

Functional annotation of identified proteins was based on sequence similarity searches carried out with Diamond BLASTP (v0.8.22.84) [10] against the Uniref50 [46] database (release August 24, 2018). The following parameters were applied: top five hits, *e*-value threshold 10, and percentage identity above 50%. The GOSlim terms (release January 30, 2017) associated with the Uniprot accession number were retrieved for each protein. KEGG annotation was performed using the GhostKOALA [31] web server. Peptides identified at FDR 1% were functionally annotated using the Unipept [43] desktop application version 1.2.1, activating the “equate I and L” and “advanced missing cleavage handling” options. Unipept peptide taxonomical information was used to calculate kingdom and phylum abundances.

## Data Availability

The mass spectrometry proteomics data have been submitted to the ProteomeXchange Consortium via the PRIDE partner repository under dataset identifier PXD026798 and project DOI 10.6019/PXD026798.

## Abbreviations

NCBInr: National Center for Biotechnology Information non-redundant
PSMs: Peptide-to-spectrum matches
FDR: False discovery rate
MAGs: Metagenome-assembled genomes
CDS: Protein-coding sequence
MF: Molecular function
BP: Biological process
GO: Gene Ontology
EC: Enzyme commission
KO: KEGG Orthology
